# Elementary school students’ motivations for responding without prejudice: the role of the student-teacher relationship

**DOI:** 10.1007/s11218-026-10196-z

**Published:** 2026-03-16

**Authors:** Teuntje van Heese, Jochem Thijs

**Affiliations:** 1 Department of Education, Utrecht University, Martinus J. Langeveld Building, Heidelberglaan 1, room E3.36, Postbox 80140, 3584 CH Utrecht, The Netherlands; 2 ERCOMER/Department of Interdisciplinary Social Science, Utrecht University, Sjoerd Groenman Building, Padualaan 14, Utrecht, 3584 CH The Netherlands

**Keywords:** Motivation, Prejudice, Student-teacher relationship, Self-determination theory, Attachment

## Abstract

Despite extensive research on prejudice reduction in schools, the motivational processes underlying students’ regulation of prejudice remain relatively understudied, even though they may provide a novel perspective for improving intergroup attitudes. The present study addressed this by examining the role of teachers in elementary school students’ internal and external motivations to respond without prejudice (RWP), building on insights from Self-Determination Theory and the so-called extended attachment perspective. Specifically, this cross-sectional questionnaire study investigated how students’ perceptions of the student-teacher relationship and teachers’ perceived anti-prejudice norms were associated with RWP motivations among both ethnic minority and majority students. The data were collected among 1101 ethnic majority and minority students (80.2% majority, 51.3% girls, *M*age = 9.8 years, *SD* = 0.5) from 59 classrooms in the Netherlands using self-report questionnaires, and analyzed with multilevel regression models. Results showed that closeness and conflict were associated with, respectively, stronger and weaker internal RWP motivations. Negative expectations were associated with a stronger external RWP motivation. These findings were consistent for both ethnic majority and ethnic minority students. The teachers’ anti-prejudice norm also did not seem to alter the associations between relational quality and RWP motivations. Hence, our findings suggest that regardless of the classroom environment, student-teacher relationships may be a valuable tool for promoting intergroup relations.

**Supplementary Information:**

The online version contains supplementary material available at 10.1007/s11218-026-10196-z.

## Introduction

There has been extensive research on how schools could contribute to prejudice reduction and positive intergroup relations among their students (see meta-analysis by Ülger et al., 2018). However, this research has largely focused on behavioral and attitudinal outcomes, while paying comparatively little attention to the motivational processes underlying the regulation of prejudice (Legault et al., 2011). Humans have automatic tendencies to be prejudiced (Devine, 1989), which appear to emerge from early childhood onwards (Dunham et al., 2013). Similar to adults (Plant & Devine, 1998), school-age children can have internal and external motivations to regulate or suppress this prejudice, with different and sometimes conflicting implications for their ethnic outgroup attitudes and intergroup relations (Hughes et al., 2016; Thijs et al., 2023). Thus, it matters why students want to regulate their prejudice. Therefore the present study aimed to identify school conditions that stimulate the “right reasons” for doing so.

Specifically, the present study had three main objectives, building on the gap described above. First, we aimed to examine whether and how perceptions of student-teacher relationship were related to elementary school students’ motivation to respond without prejudice (RWP)^1^. Second, we investigated whether teachers’ anti-prejudice norms moderated these associations. Third, we went beyond previous research by exploring whether RWP motivations and their associations with the student-teacher relationship differ among ethnic minority and majority students.

In doing so, our overarching goal was to identify relational factors within the classroom that can inform interventions aimed at fostering positive intergroup relations in schools.

We used cross-sectional survey data, collected in in Dutch elementary schools (Grades 3–6), where individual teachers are especially important to students as they tend to have one or two of them the whole year round.

### Prejudice regulation and motivation

According to Devine’s (1989) seminal prejudice model, there is an important difference between having group-based associations and the personal endorsement of prejudice. From a young age, individuals are exposed to group stereotypes which, once internalized, can automatically affect their thoughts, feelings, and behaviors toward outgroup others. However, they can also regulate and inhibit this automatic response, which means that (implicit) stereotype knowledge does not inevitably lead to (explicit) prejudice (Devine, 1989). Although Devine’s model is not a developmental one, it can be well applied to school-age children. Research has shown that the automatic (or implicit) prejudice it assumes emerges from a very early age (Dunham et al., 2008) and remains invariant from childhood to adulthood (Dunham et al., 2013). Moreover, children as young as six have been found to hide their group biases from others (de França & Monteiro, 2013; Rutland et al., 2005), indicating at least a rudimentary form of prejudice regulation.

Because prejudice regulation takes energy, people need to be motivated for it. Plant and Devine (1998) made the empirical distinction between an internal RWP motivation, which involves the personal conviction that prejudice is wrong and should not be expressed, and an external RWP motivation, which indicates the desire to conform to anti-prejudice norms in order to avoid social scorn and disapproval. Several studies have examined these motivations among adults, and whereas the internal motivation typically shows a negative relation to people’s privately held prejudice, the external one has been found to be unrelated or sometimes even positively related to it (Bamberg & Verkuyten, 2022; Plant et al., 2003; Plant & Devine, 1998). Moreover, whereas the former has been associated with less anxiety in intergroup interactions, the latter has been associated with more of it (Butz & Plant, 2009). Thus, the external motivation is less effective than the internal one and sometimes even counterproductive, presumably because the pressure to avoid social rejection creates backlash through feelings of threat and frustration (Plant, 2004; Plant & Devine, 2001). Two recent studies showed that the two RWP motivations are important for elementary school students as well (Hughes et al., 2016; Thijs et al., 2023). The first study, conducted among White students in the United States, found that internal RWP motivation was positively related to children’s outgroup attitudes and negatively related to outgroup anxiety and bias. External motivation was associated with reduced bias, but showed no relation to outgroup attitudes and was positively related to outgroup anxiety (Hughes et al., 2016). The second study used the dataset for the present research and included ethnic Dutch majority students only. It showed that children’s positive outgroup attitudes were positively related to internal RWP motivation and negatively to external RWP motivation (Thijs et al., 2023). In both studies, the two motivations were relatively independent, just as in the research among adults.

The conclusion that both RWP motivations have different effects – in adults as well as children – is fully in line with Self-Determination Theory (SDT; Deci & Ryan, 1985; Ryan & Deci, 2000), and some studies have even directly used the theory’s taxonomy to study the regulation of prejudice (Legault et al., 2007; Legault & Green-Demers, 2012; Thijs et al., 2016). SDT can be considered a macro-theory of human motivation and states that behavior is more effective when it is self-determined, that is, when it is regarded as personally important and experienced as originating from within (autonomous motivation) (Deci & Ryan, 1985; Ryan & Deci, 2000). As the internal RWP motivation involves one’s personal beliefs and values, it implies a strong self-determination. However, the external motivation clearly implies a lack of it, as the regulation of prejudice is controlled by concerns about negative reactions from others (controlled motivation) (see Legault et al., 2007).

### Student-teacher relationships and RWP motivations

In the present study, we combined SDT with the so-called extended attachment perspective on the student-teacher relationship (Bergin & Bergin, 2009; Verschueren & Koomen, 2012), to examine the links between the latter and students’ RWP motivations. According to SDT, the experience of self-determination depends on the satisfaction and the frustration of three basic psychological needs, including the need for relatedness. The theory claims that people are more likely to pursue what they find inherently interesting and valuable if they feel connected to important others, and states that this makes them more prone to internalize the values and standards of those others as well (Deci & Ryan, 2000; Vansteenkiste et al., 2020). However, feeling rejected by others (need frustration) would make this less likely, and would rather make people inclined to be externally motivated and do things because others expect them to (Haerens et al., 2015; Olafsen et al., 2021). SDT has been extensively applied to the context of education (see Howard et al., 2021) and there are clear indications that teachers can promote and facilitate students’ self-determined motivation or school engagement and achievement (outcomes of this motivation) by fulfilling versus frustrating their needs for relatedness (for meta-analyses, see Roorda et al., 2011; Vasconcellos et al., 2020).

The extended attachment perspective also maintains that teachers are important for the motivations of their students. It states that teachers can function as ad hoc attachment figures to school-aged children, providing them with a “secure base”, from which to actively engage with the world, and a “safe haven”, to return to in times of stress (Ainsworth, 1989; Verschueren & Koomen, 2012). Thus, secure relationships with their teachers enable students to follow their natural inclinations to discover and learn (Pianta et al., 2003), and allow them to be self-reliant and independent (Bergin & Bergin, 2009). The security in the student-teacher relationship is typically assessed via the dimension of *closeness*, which involves the degree of mutual warmth and trust. However, the student-teacher relationship can also be characterized by insecurity (Bergin & Bergin, 2009), which can interfere with students' engagement and performance (see Roorda et al., 2011). According to the attachment perspective, there are two types of this insecurity that are indicated by the dimensions of *conflict* and *dependency*. Conflict refers to the experience of mutual negativity and discord, and dependency involves the degree to which the child is overreliant on the teacher and excessively concerned with their availability. As with closeness, both dimensions can be identified in students’ relationship perceptions, although students dependency-related worries seem to be part of a broader dimension that involves uncertainty about the teacher more generally, and which is therefore labeled *negative expectations* (de Jong et al., 2018; Koomen & Jellesma, 2015).

**Fig. 1:**
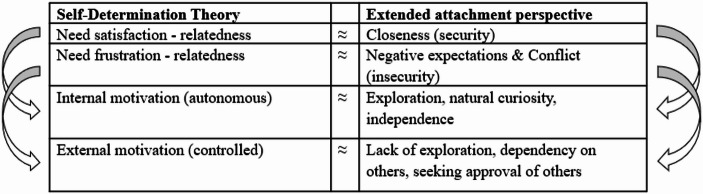
Proposed theoretical framework integrating self-determination theory and the extended attachment perspective on the student–teacher relationship

Theoretically, the experiences of security and insecurity in the student-teacher relationship correspond to, respectively, the satisfaction and frustration of the need for relatedness as posited by SDT. However, the attachment perspective complements SDT in two ways, and thereby provides additional reasons to expect that student-teacher relationships are relevant for students’ RWP motivations. First, it further explicates why student-relationship may matter for motivations regarding outgroup attitudes specifically. Attachment theory (Bowlby, 1982) states that there is an innate fear and distrust of strangers, but also that this fear can be mitigated by the experience of relational security. Outgroup others can be relatively unfamiliar and therefore perceived as threatening (Turner et al., 2007). Accordingly, findings from several studies suggest that a sense of secure attachment can result in a more open approach toward them (e.g., Boag & Carnelley, 2016; de Bruijn et al., 2022; Geerlings et al., 2017; Miklikowska et al., 2019; Mikulincer & Shaver, 2001), whereas insecure attachment can have negative effects on this (di Pentima & Toni, 2009; Hofstra et al., 2005; Mikulincer & Shaver, 2001; Van Oudenhoven & Hofstra, 2006). Second, the extended attachment perspective broadens the conceptualization of unfavourable student-teacher relationships, making their links to external motivation more evident. It suggests that relational need frustration can take on different forms, with potentially different motivational implications. Unlike the dimension of conflict, the dimension of negative expectations involves students excessive concerns with teacher availability and approval. These negative expectations might be especially relevant to students’ external RWP motivation, as the latter implies a drive to seek approval and avoid negative feedback from others (cf., Thijs & Fleischmann, 2015). The integration of SDT and the extended attachment perspective is reflected in Fig. 1. Based on this proposed integrated theoretical framework, we formulated three hypotheses. We expected that closeness would be associated with a stronger internal RWP motivation (H1), and that conflict and negative expectations would be associated with a stronger external RWP motivation (H2). Moreover, we hypothesized that the latter relation would be most pronounced for negative expectations (H3). We controlled for gender and age in testing these hypothesized relationships, as previous research found that girls experience more closeness and less conflict in the relationship with their teacher (Koomen & Jellesma, 2015; Thijs & Fleischmann, 2015), and that external and internal RWP motivations are generally stronger among older children (Hughes et al., 2016).

### The moderating role of teachers’ anti-prejudice norms

Several studies suggest that teachers can reduce the prejudice of their students by emphasizing norms opposing prejudice or advocating values such as equality, egalitarianism, and multiculturalism (e.g. Konings et al., 2021; Schwarzenthal et al., 2018). While SDT and the attachment perspective complement each other in their theoretical expectations for how students’ relations with their teacher could affect their RWP motivations, they would differ in their hypotheses regarding the role of teachers’ anti-prejudice norms. This difference stems from the fact that both theories address somewhat different underlying processes for the association between relatedness and internal motivation.

SDT claims that a sense of relatedness not only directly facilitates the experience of intrinsic motivation to discover and interact with the unknown, but also promotes a process of internalization whereby individuals adopt the standards of those they feel related to (via increasing their identified and integrated regulation) (Deci & Ryan, 2000; Ryan & Deci, 2000). This implies that the association between student-teacher relational closeness and students’ internal RWP motivation would be stronger if their teacher endorses a norm against prejudice. Conversely, SDT states that individuals only superficially comply with the standards of important others if those others frustrate their need for relatedness. Thus, if students’ teacher stresses the normative unacceptability of prejudice, their negative expectations and experience of conflict would be more strongly related to their external RWP motivation. Again, this may especially be true for negative expectations, as a strong anti-prejudice norm from the teacher could prompt students who are particularly focused on gaining their teacher’s approval, to regulate their prejudice to secure this approval. By contrast, the extended attachment perspective would claim that the anticipated effects of the student-teacher relationship would be relatively independent of the teacher’s anti-prejudice norm, or other teacher characteristics for that matter. The experiences of relational security or insecurity themselves would be enough to, respectively, promote or undermine openness to outgroup others (see de Bruijn et al., 2022; Geerlings et al., 2017).

Considering the different possibilities suggested by both theoretical approaches, we explored without specific hypotheses whether the expected relationships between the student-teachers relationship dimensions and the internal and external RWP motivation would be more pronounced for teachers who, according to their students, more strongly expressed an anti-prejudice norm. We also controlled for the ethnic composition of the classroom, as such norms tend to be more prevalent in ethnically diverse classrooms (Vervaet et al., 2018).

### Majority and minority students

To the best of our knowledge, prior research on RWP motivations (in children as well as adults) has predominantly addressed ethnic or racial majority groups. This focus is understandable. Ethnic and racial minority groups have historically been disadvantaged due to the existence of racist hierarchical structures with majority groups in position of power and privilege (Salter, 2018), and accordingly, prejudice among the latter constitutes a considerably larger problem than prejudice among the former. Still, there are good reasons to study RWP motivations and their associations with the student-teacher relationship in *both* majority *and* minority students. First, research has shown that both groups can be prejudiced, even though majority group children are typically more biased or prejudiced than their minority peers (e.g., Leman et al., 2013; Pektas et al., 2023). Second, many elementary school students receive some form of diversity education and are thus exposed to the notion that prejudice is morally wrong and socially undesirable. Accordingly, prejudice regulation will be important regardless of children’s ethnic or racial background, and for intervention purposes, it is essential to examine whether their underlying motivations are comparable. Third, educational researchers have shown that, even though minority students sometimes share less favorable relationships with their teachers compared to their ethnic majority classmates (e.g., Thijs & Fleischmann, 2015; see also Steenwegen et al., 2024), these relationships can actually be more important for their motivation and school adjustment (Den Brok et al., 2010; Roorda et al., 2011).

Thus, to better understand prejudice regulation in today’s increasingly diverse classrooms, and to further enhance the external validity of RWP motivation research more generally, we examined whether the internal and external RWP motivations could be differentiated among ethnic minority students as well, and explored whether these motivations were similarly associated with the student-teacher relationship across minority and majority students.

**Fig. 2:**
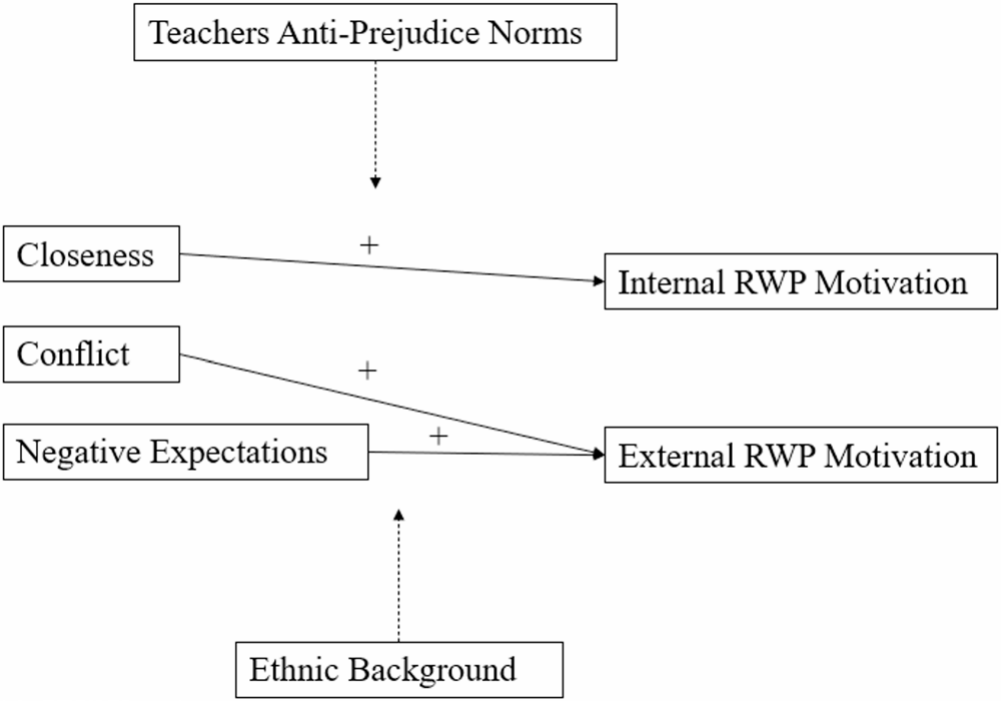
Proposed conceptual model depicting hypothesized and exploratory relations among key variables. *RWP* responding without prejudice. Dashed lines indicate exploratory hypotheses

Figure 2 provides an overview of the central proposed relationships examined in this study. The solid lines reflect the three main expectations, and the dashed ones the moderation effects that were explored.

## Methods

### Participants and procedure

The present research was part of a larger research collaboration on teachers’ dealings with diversity which was approved of by the Ethics Review Board of the University of Amsterdam (project no. 2017-CDE-8653). The data were collected in three waves during the 2017–2018 school year, of which only the first wave was used in the present study. The original group of participants were 1442 Grade 3–6 students and 59 of their (main)^2^ teachers, from 32 primary schools in the Netherlands. Students completed a pen-and-paper survey in their classroom under supervision of a researcher or research assistant, and informed passive parental consent was obtained for each of them. All participation was voluntary and participants could stop at any time.

For the current study, we only used the student questionnaire data. We selected students who could be either identified as ethnic Dutch – henceforward referred to as “majority” – or as Turkish, Moroccan or Surinamese^3^ – henceforward referred to as “minority” – although we used the original sample to calculate the ethnic composition of the classroom (see below for measures and exact selection criteria). This selection resulted in a final sample of 1101 students (883 majority, 218 minority) in 59 classes. The participants were seven to thirteen years old (*M* = 9.80, *SD* = 1.20) and their gender was approximately equally distributed (51.3% girls).

For the selected student sample, less than 1.6% of the scores on the student-teacher relationship and RWP motivation measures were missing. Little’s MCAR test indicated χ²(11177) = 2067.63, normed χ² = 0.19, suggesting that data are likely missing completely at random (MCAR) (Ullman, 2001). To deal with these missing student data and preserving information for scale averages, multiple imputation on the item level was used in IBM SPSS Statistics 28.0 (IBM,^2021^). We requested 50 imputed files (Jakobsen et al., 2017) for the items of the student-teacher relationship and RWP motivation measures (and also for their individual perceptions of the teacher norm; see below). As auxiliary variables, we included the age and gender of the students reported at Wave 1, as well as dummies for the classes in which they were nested (Drechsler, 2015).

### Measures

#### Ethnicity and ethnic classroom composition

At each wave, students were asked to indicate their own ethnicity and their mother’s and father’s ethnicity, after reading the following introduction: “In the Netherlands, there are different cultural groups. Those groups have to do with the country where people or their families come from. For example, you have Turks, Moroccans, Surinamese, but also Dutch and many more.”. The response categories were Turkish, Moroccan, Surinamese, Dutch, and “other” (an open answer category). Students could select multiple categories. For the present study, we regarded children as ethnic Dutch if they identified themselves, their mother, *and* their father as Dutch only^4^, and as Turkish, Moroccan, or Surinamese if they identified at least one of their parents as belonging to only one of those groups. We also created a measure for ethnic classroom composition, based on the information of the original sample, by calculating the proportion of ethnic Dutch students in each class (*M* = 0.631, *SD* = 0.314, *N* = 59). A lower score on this measure reflected a larger share of non-Dutch students.

#### Student-teacher relationship

Students’ perceptions of the student-teacher relationship were measured with a shortened version of the Student Perception of Affective Relationship with Teacher Scale (SPARTS, Koomen & Jellesma, 2015). This version contained six-item subscales for *closeness* (e.g., “I tell my teacher things that are important to me”, “The teacher usually knows how I feel”), *negative expectations* (e.g., “I don’t like it when my teacher pays attention to other children”, “I wish my teacher had more time for me”) and *conflict* (e.g., “I feel my teacher doesn’t trust me”, “I can be very angry with my teacher”) (see Table S1 in the Supplementary Materials for all items). The response scale ranged from 1 (*No*,* that is not the case*) to 5 (*Yes*,* that is the case*). Analyses of internal consistency across the imputed datasets yielded Cronbach’s alphas ranging from, respectively, 0.732 to 0.739 for closeness, 0.668 to 0.672 for negative expectations, and 0.736 to 0.740 for conflict. As described in the results section, we also examined the factor structure underlying these measures.

#### Motivations to respond without prejudice

Students’ motivations to respond without prejudice were assessed with a new instrument based on internal and external RWP motivation questionnaires for adults (Legault et al., 2007; Plant & Devine, 1998). Students were asked about their endorsement of different reasons for “acting nice towards children from other countries or cultures” on a scale from 1 (*No!*) to 5 (*Yes!*) (see Table S1 in the Supplementary Materials for all items). Following Legault et al. (2007), we operationalized internal RWP motivation not only as motivation based on personal beliefs and attitudes, but also as intrinsic motivation, that is, engaging with outgroups for the inherent enjoyment of it. This aligns the measure more closely with SDT, in which intrinsic motivation is a core component of internal (or self-determined) motivation (Ryan & Deci, 2000). Unlike other RWP measures (including Hughes et al., 2016), the new instrument did not refer to “prejudice” or “stereotypes”, as these terms were considered too abstract for children. Moreover, it used a positive formulation (“acting nice”), as indicating disagreement with negative formulations (e.g., “not being mean”) can be complex and confusing for children (Marsh, 1986). Although acting nice is not the same as not showing prejudice, it does imply it and childhood prejudice often manifests as a lack of positive outgroup behavior (Rutland et al., 2007). The instrument includes an internal motivation scale consisting of six items (e.g., “because I think it is wrong to act mean to them”, “because I like to”), and an external motivation scale consisting of four items (e.g., “because other people tell me to”, “because otherwise people might think I am a bad child”). Earlier research found these scales to be, respectively, positively and negatively related to the ethnic outgroup attitudes of the majority students in the present sample (Thijs et al., 2023). Across the imputed datasets, Cronbach’s alpha ranged from 0.748 to 0.752 for the internal motivation, and from 0.798 to 0.803 for the external motivation. The factor structure behind these measures is described in the results section.

#### Teachers' anti-prejudice norms

Students were presented with a neutral doodle character who expressed the following three statements in a speech balloon: “You should be nice and honest to people from other cultures,” “It is wrong to be mean to people from other countries,” and “People from all cultural groups are equal.”, which were based on research on multicultural education in the Netherlands (Verkuyten & Thijs, 2013). After each statement, students were asked to indicate how often, respectively, their teacher, classmates, and parents uttered it. The response scale ranged from “Absolutely never!” (1) to “Very very often!” (5). For the present study we only used the teacher items, and we averaged perceptions of all participating students per classroom, to obtain a relatively objective assessment of the anti-prejudice norm of each teacher. First, we calculated individual scale scores by taking the mean of the three items for all students without missing scores on them (*N* = 1367; Cronbach’s alpha = 0.814). Next, we examined whether we could aggregate these scores per classroom by calculating two intraclass correlations (ICC’s). The first, ICC1, indicated the degree to which classmates’ perceptions were consensual, and the second, ICC2, indicated the degree to which the individual perceptions within each classroom were consistent between students (see Lüdtke et al., 2009). ICC1 was 0.238 which means that 23.8% of the variance in students’ perceptions existed at the level of the classroom, and hence the teacher. ICC2 was 0.879 suggesting high consistency between classmates. Thus, we averaged the individual perceptions per classroom.

### Data analytic strategy

For our main analyses, we tested several multivariate multilevel models in Mplus version 8.7 (Muthén 1998–2017), in which we specified two levels: the student level, and the classroom level. We used the deviance test (Δ − 2LL) and both the Akaike and Bayesian information criteria to evaluate model improvement (Hox et al., 2018). To avoid estimation problems based on having more parameters than clusters, the models included observed measures instead of latent variables. However, we first examined the factor structure behind the relationship and motivation scale items in the original (non-imputed) data, and tested whether it was configurally, metrically, and scalarly equivalent for majority versus minority students. To avoid having more parameters than clusters, the classroom clustering was not considered in those confirmatory factor analyses. The remaining analyses were conducted on the imputed datasets. All models were estimated with MLR to account for possible non-normality in the data.

## Results

### Preliminary results

#### Factor structure and measurement invariance

First, a set of multiple group Confirmatory Factor Analyses (CFAs) were conducted in Mplus, to investigate the factor structure behind our measures of the internal RWP motivation, the external RWP motivation, closeness, negative expectations, and conflict, and to examine whether this structure was comparable for majority versus minority students. Fit statistics are shown in Table 1. We opted for a five-factor model, ensuring that the constructs are empirically distinct while accounting for measurement error (Kline,^ 2011^). To further verify that the fit of one scale was not compensating for another, we also tested two separate CFA’s. The results were comparable with the five-factor model and are presented in the Supplementary Materials (Table S2 and Table S3).

We started with a configural model with five factors corresponding to each of the measures and without any cross-loadings. After error correlations were allowed between two internal motivation items and between two negative expectations items, the fit of this configural model was acceptable (i.e., CFI and TLI > 0.90, RMSEA and SRMR < 0.10; see Kline, 2011). Next, we specified a metric version of this model by constraining the factor loadings for both groups, and a scalar one by constraining the factor loadings and the intercepts for both groups. As shown in Table 1, the Satorra-Bentler scales χ^2^ difference test indicated no difference between the configural and the metric model but a significant difference between the metric and the scalar model. However, as χ^2^ difference tests are extremely sensitive in relatively large samples, we relied on CFI and RMSEA differences (Putnick & Bornstein, 2016). These respective differences were only smaller than 0.005 and 0.01 in the first comparison, indicating that metric, but not scalar invariance could be assumed for our sample with unbalanced groups (Chen, 2007). As a result, the direct effects of ethnic background, operationalized as a dummy-coded variable, should be interpreted with caution, as they effectively reflect comparisons of latent means. However, metric invariance permitted meaningful comparisons of associations between relationship variables and RWP motivations across majority and minority students, as well as the use of ethnic background as a moderator (Cheung & Lau, 2011).

**Table 1:** Results of multiple group confirmatory factor models

	χ^2^	df	Δ χ^2^	Δdf	RMSEA (90% CI)	SRMR	CFI	TLI
Configural model	1118.538	624			0.038 (0.034–0.042)	0.051	0.912	0.901
Metric model (equal factor loadings)	1136.700	646	23.329	22	0.037 (0.034–0.041)	0.053	0.912	0.905
Scalar model (equal factor loadings and intercepts)	1210.275	668	79.139**	22	0.038 (0.035–0.042)	0.055	0.903	0.898

Error correlations allowed between two internal motivation items and between two negative expectation items. Δ χ^2^ based on the Satorra-Bentler scaled χ^2^ difference test. ** *p* < 0.01

#### Means and intercorrelations

The means and intercorrelations for all variables in this study are included in Table 2. On average, students reported relatively strong internal RWP motivations and high levels of closeness, and relatively weak external RWP motivations and low levels of negative expectations and conflict. Besides, conflict showed a considerable relation to closeness (*r* = −0.448) and negative expectations (*r* = 0.454). The two RWP motivations were weakly correlated themselves, and differentially related to the relationship variables: whereas closeness and conflict were associated with, respectively, a stronger and a weaker internal motivation, negative expectations was associated with a stronger external motivation. In addition to this, the correlations revealed some small differences between majority versus minority students. The former reported a weaker internal RWP motivation, as well as more closeness and less conflict and negative expectations. Students in classrooms with a larger share of majority students also had generally less internal RWP motivation, and the collectively perceived teacher norm was positively related to the internal motivation and negatively to the external motivation – although these correlations should be inspected with care as they were uncorrected for the multilevel structure of the data.

Finally, Table 2 indicated that it was meaningful to control for gender and age. Girls had on average stronger internal RWP motivations than boys and tended to experience more closeness and less conflict in the relationship with their teacher. Older students reported a weaker external RWP motivation, and more closeness and less negative expectations than their younger peers.

**Table 2:** Intercorrelations and means

	1	2	3	4	5	6	7	8	9	M	SD	ICC
1. Internal RWP motivation										3.962	0.751	0.027
2. External RWP motivation	0.077*									2.277	1.033	0.066
3. Closeness	0.226**	−0.057								3.835	0.737	0.076
4. Negative expectations	−0.010	0.204**	−0.243**							1.699	0.648	0.057
5. Conflict	−0.154**	0.058	−0.448**	0.454**						1.499	0.596	0.048
6. Majority vs. minority	−0.135**	−0.035	0.067*	−0.075*	−0.077*					0.302	0.399	
7. Gender (girl vs. boy)	0.187**	−0.053	0.148**	0.058	−0.186**	−0.032				0.014	0.500	
8. Age	−0.25	−0.128**	0.116**	−0.177**	0.008	0.074*	−0.042			9.803	1.201	
9. Teacher norms	0.125**	−0.060*	0.041	−0.054	0.015	−0.437**	−0.022	0.449**		2.503	0.535	
10. Ethnic composition	−0.141**	−0.032	0.046	−0.084**	−0.070*	0.800**	−0.029	0.100**	−0.509**	0.665	0.302	

* *p* < 0.05, ** *p* < 0.01. *M* mean, *SD* standard deviation, *ICC* intraclass correlation coefficient, indicating the proportion of total variance attributable to between-group differences. Means, correlations and the ICC’s were pooled across 50 imputed datasets, the standard deviations represent the average across imputations

### Main analyses

Next, we tested several multivariate multilevel models to examine and explain the variation in students’ RWP motivations within (Level 1), and between classrooms (Level 2). In these models, the two motivations were simultaneously included as correlated dependent variables. Model fit statistics are provided in Table S4 of the Supplementary Materials.

First, we tested an intercept-only model to examine how the variance in both measures was distributed across the two levels. This model yielded an ICC1 of 0.027 (*p* < 0.05) for the internal motivation and an ICC1 of 0.065 (*p* < 0.01), indicating that, respectively, 2.7% and 6.5% of the variance in these measures existed at the classroom level.

In a second model, we added the three relationship measures, as well as group (majority versus minority), gender, and age as predictors at Level 1, and teachers’ anti-prejudice norms and ethnic composition at Level 2. The results of this second model are shown in Table 3. The model fit significantly improved (*p* < 0.001), and consistent with the bivariate correlations in Table 2, closeness and conflict were respectively positively and negatively related to students’ internal RWP motivation, and negative expectations were associated with a stronger external RWP motivation. Moreover, there were no effects of ethnic group and age, but girls had a stronger internal and a weaker external RWP motivation. At the classroom level, the teacher’s anti-prejudice norm was positively related to students’ internal RWP motivation.

**Table 3:** Multilevel regression models predicting internal and external motivation to respond without prejudice

	Internal motivation		External motivation	
	b (SE)	β (SE)	b (SE)	β (SE)
Level 1				
Closeness	0.187 (0.036)**	0.186 (0.35)**	0.001 (0.052)	0.001 (0.038)
Negative expectations	0.037 (0.044)	0.033 (0.038)	0.329 (0.062)**	0.212 (0.040)**
Conflict	−0.098 (0.047)*	−0.079 (0.038)*	−0.100 (0.075)	−0.059 (0.044)
Majority vs. minority	−0.126 (0.083)	−0.068 (0.044)	−0.036 (0.129)	−0.014 (0.051)
Gender	0.213 (0.044)**	0.144 (0.030)**	−0.154(0.064)*	−0.077 (0.032)*
Age	−0.050 (0.026)	−0.081 (0.042)	−0.022 (0.043)	−0.026 (0.052)
Level 2				
Teachers’ norms	0.158 (0.058)**	0.686 (0.267)**	−0.134 (0.098)	−0.362 (0.219)
Ethnic composition	−0.071 (0.118)	−0.175 (0.277)	−0.147 (0.162)	−0.225 (0.242)
Variance (% explained)				
Level 1	0.496 (9.7%)		0.969 (2.6%)	
Level 2	0.006 (60.0%)		0.037 (47.9%)	

* *p* < 0.05, ** *p* < 0.01

Next, we examined whether the effects of the relationship measures differed for majority versus minority students, by adding interactions between these measures (mean-centered) and the ethnic group contrast as predictors to the second model. The model fit did not improve and none of these interactions were significant (*p* > 0.10), see Table S5 of the Supplementary Materials).

Lastly, we tested whether the effects of the relationship measures in our second model depended on the collectively perceived teacher norm. In doing so, we first randomized the slopes of the relationship measures at the classroom level. All slopes were nonsignificant (*p* > 0.20, see Table S6 of the Supplementary Materials, Model 4), indicating similar effects across classrooms. Consistent with this, the model fit did not improve. Still, as the nonsignificance of random slopes does not exclude the possibility that specific higher-level variables moderate lower-level relationships (Snijders & Bosker, 1999), we proceeded by entering the cross-level interactions between the relationship variables and the perceived teacher norm. As shown in Table S6 of the Supplementary Materials (Model 5), only the cross-level interaction with negative expectations on internal RWP motivation was significant, *b* = −0.200, *SE* = 0.70, *p* = 0.003. This interaction suggests that students with negative expectations have a stronger internal RWP motivation if their teacher does not strongly endorse an anti-prejudice norm. However, as the model fit for this latter model worsened on all three indicators (see Table S2), this result should be interpreted with care (Hox et al., 2018).

## Discussion

Primary schools are important contexts for countering ethnic prejudice and promoting positive intergroup relations. Research suggests that students’ motivations to respond without prejudice (RWP) may play a key role in this, but to date these motivations have received little theoretical and empirical attention in the educational literature. Responding without prejudice requires effort, and is more effective when driven by internal motivations based on personal beliefs than by external motivations based on concerns about social disapproval (Bamberg & Verkuyten, 2022; Hughes et al., 2016; Plant et al., 2003; Plant & Devine, 1998). Thus, it is important to attend to these motivations in school-age children, and to examine the factors that contribute to each of them. The present study did so by considering students’ perceptions of the student-teacher relationship as well as the moderating role of teachers’ anti-prejudice norms, including both ethnic majority and minority students.

### The importance of the student-teacher relationship

Firstly, we evaluated three hypotheses based on the combination of Self-Determination Theory (SDT; Deci & Ryan, 1985; Ryan & Deci, 2000) and the extended attachment perspective on the student-teacher relationship (Verschueren & Koomen, 2012). Our first expectation involved a positive link between students’ closeness with their teacher and their internal RWP motivation and our results clearly confirmed this. This finding can be well situated within the extended attachment perspective, where relational security, characterized by mutual positivity and warmth, yields a “secure base” for students to explore and engage openly with the world, including “unfamiliar” others (Mikulincer & Shaver, 2001; Boag & Carnelley, 2016). In this sense, feeling securely connected to the teacher may nurture the genuine, curiosity-driven interest towards others that is central to internal RWP motivations (see Legault et al., 2007). Additionally, from a SDT perspective, the experience of a warm and supportive relationship implies that one’s need for relatedness is satisfied, which generally promotes more self-determined, internal motivations (Deci & Ryan, 1991; Ryan & Deci, 2017).

Our second and third hypotheses pertained to conflict and negative expectations. These relationship dimensions correspond to the concepts of need frustration in SDT and relational insecurity in the extended attachment perspective, and we expected them to be positively related to students’ external RWP motivation. Moreover, drawing on the extended attachment perspective, we expected this relation to be most pronounced for negative expectations, given that students holding such expectations are preoccupied with the teacher’s availability and approval (Koomen & Jellesma, 2015). These hypotheses were partially supported. Although we did not find a significant relationship for conflict, students who had more negative expectations of their teacher were indeed more externally motivated to respond without prejudice. In addition to this, we obtained a negative relation between conflict and the internal RWP motivation. We had no a priori hypothesis for this finding, but it was still in line with SDT’s principle that frustration of the need for relatedness hinders a self-determined motivation (see e.g., Vasconcellos et al., 2020).

Taken together, the present results align well with previous research demonstrating that that experiences of relational security and relational insecurity have, respectively, positive and negative implications for outgroup attitudes (e.g., Boag & Carenelly, 2016; de Bruijn et al., 2022; di Pentima & Toni, 2009; Hofstra et al., 2005; Mikulincer & Shaver 2001; Van Oudenhoven & Hofstra, 2006). Our findings extend this by showing how these relational dynamics specifically shape the type of RWP motivation students develop, which are crucial for understanding prejudicial responses (Bamberg & Verkuyten, 2021). Moreover, our findings cannot be attributed to the interrelations of the three relationship variables. In line with other studies (e.g., Koomen & Jellesma, 2015; Thijs & Fleischmann, 2015), we found that conflict was considerably related to closeness and negative expectations. Yet, we tested the independent effects of these variables. In fact, their effects in the multilevel regression analyses fully corresponded to their bivariate correlations with the RWP motivations. This indicates, for example, that the negative link between conflict and the internal RWP motivation does not merely exist in the rather “artificial” situation where closeness and negative expectations do not matter, and that the three relationship measures have qualitatively different implications for prejudice regulation in students.

### The role of teachers’ anti-prejudice norms

Secondly, we examined whether the associations between students’ relationship perceptions and their RWP motivations were moderated by the collectively perceived anti-prejudice norm of their teacher. We did not formulate explicit hypotheses regarding this moderation, as the two theoretical perspectives guiding our study yielded diverging predictions.

Overall, there was little evidence that teachers’ anti-prejudice norms moderated the effects of the relationship measures. We only observed a negative interaction for negative expectations, suggesting that negative expectations were associated with a stronger internal RWP motivation in the absence of an anti-prejudice norm. However, due to statistical concerns (a deterioration of model fit), this result is not considered robust enough to draw any conclusions about. Rather, our results indicate that the links between relationship quality and RWP motivations were not amplified in classrooms where teachers were perceived as strongly emphasizing the unacceptability of prejudice. Hence, we found no support for an SDT-based mechanism in which students with more positive student-teacher relationships more strongly internalize their teachers’ norms, while those with negative relationships merely comply with them in a controlled way. Moreover, relationship effects did not differ across classes, suggesting that they were also not contingent upon other teacher characteristics not included in the present study. Thus, overall, these results indicate that students’ sense of relational security or insecurity alone may be sufficient to promote or hinder a self-determined motivation to regulate prejudice.

Nonetheless, teachers’ anti-prejudice norms showed a positive main effect on students’ internal RWP motivation (see also Thijs et al., 2023). This implies that such norms may operate through an independent pathway, rather than through interactions with the student-teacher relationship. One possibility is that anti-prejudice norms operate as social norms, feeling as a shared group opinion based on mutual agreement and commitment instead of individual pressure (Tomasello & Vaish, 2013). Therefore, teachers’ norms might shape a shared anti-prejudice climate with which students personally identify, resulting in stronger internal RWP motivations.

A further possibility is that certain moral values may directly resonate intrinsically with students. Research suggest that children have internal concepts of fairness and equity that guide how they believe others should be treated (see Killen et al., 2012). In this view, anti-prejudice norms may not so much impose an external standard as they activate existing prosocial orientations that are already part of students’ developing moral self. Such values may be more readily integrated into the self without requiring strong need-supportive conditions, resulting in a direct effect on internal RWP motivations and highlighting a potential mechanism for intrinsic prosocial motivation that merits further study.

### Minority and majority students

Third, we explored RWP motivations and their associations with the student-teacher relationship among ethnic minority and majority students. An important finding of our study is that the internal and external RWP motivations can be reliably assessed in majority as well as minority students, allowing for meaningful comparisons of their associations with other variables, while acknowledging that scalar invariance was not achieved. For obvious reasons, the research on prejudice regulation has almost exclusively focused on ethnic or racial majority individuals. However, minority students can be prejudiced as well (see, e.g., Verkuyten & Thijs, 2010), and just as their majority peers, be exposed to the notion that the expression of prejudice is wrong and socially unacceptable. Our findings indicate that both groups were similarly concerned with regulating that expression, and therefore it is important to not exclude them from prejudice research, even though prejudice among majorities constitutes a considerably larger problem (Salter, 2018). More importantly, the links between the relationship measures and the RWP motivations were similar for both groups, despite some slight differences in perceived relationship quality. This underlines the relevance of our findings and further supports the view that the processes outlined in SDT and attachment theory represent universal principles that can be applied to different ethnic or cultural groups (Bowlby, 1982; Ryan & Deci, 2000; Vansteenkiste et al., 2020).

### Practical implications

Our findings have important implications for educational practice. Prejudice and discrimination are not only societal concerns but also have profound detrimental consequences for individual functioning. Longitudinal research shows that holding ethnic prejudice as well as being the target of prejudice and discrimination are associated with lower well-being and poorer mental health (Bobba & Crocetti, 2024; Wang et al., 2025). Given the central role of schools in students’ daily lives, schools and teachers are uniquely positioned, and have a responsibility, to address and prevent the development and perpetuation of prejudice (Ülger et al., 2018).

Yet, doing so is not always easy, especially when there is strong polarization around diversity (see, e.g., Tribukait, 2021). In those cases, teachers might be hesitant in their communications about prejudice and diversity as those communications might be criticized and regarded as contested by some students and especially their parents. Moreover, individual teachers can also have their own beliefs about “dealing with diversity” (Hachfeld et al., 2011), which do not always accord with what is expected of them. However, there is no debate about the value of high-quality student-teacher relationships, which means that they are a rather indisputable and practical tool for promoting positive intergroup relationships. The finding that the effects of closeness, conflict, and negative expectations were similar across classrooms and teachers attests to their practical significance. Apparently, the mere experience of relational security (versus insecurity) is sufficient to produce more self-determined RWP motivations, and paradoxically this means that even biased and prejudiced teachers could contribute to this. Thus, by fostering high-quality student-teacher relationships, schools may support students’ motivation to respond without prejudice, which may also contribute to broader societal goals, such as reducing inequalities (UN SDG 10,^ 2015^). Accordingly, recent studies that developed effective interventions to enhance student-teacher relationships might also yield beneficial outcomes in this regard (e.g. Bosman et al., 2021; Duong et al., 2018).

Besides, the fact that we obtained similar results for majority and minority students suggests that it is also important to foster “the right” motivation to regulate prejudice among the latter, in order to eventually promote positive intergroup relations among *all* students. Although our study focused on teachers’ relations with their students rather than their communications about prejudice and discrimination, our findings have implications for situations where teachers do want to discuss these problems. Anti-prejudice interventions in schools sometimes focus on majority groups as perpetrators of inequality and discrimination. This is understandable given the presence of systemic racism (Salter, 2018), but unfortunately, the message that they belong to the perpetrator group might undermine majority students’ sense of relatedness and thereby have negative implications for their prejudice regulation. Our findings that minority students are concerned with prejudice regulation as well suggest that prejudice is a common human problem even though in practice, some groups are considerably more likely to be victims of discrimination than others. We believe that teachers can promote positive intergroup relations among all of their students by giving this subtle message and emphasizing the related notion that prejudice and discrimination are wrong independent of the actors involved.

## Conclusion

To the best of our knowledge, this study is the first to examine the relations between the RWP motivations of both ethnic majority and minority students and their perceptions of the student-teacher relationship. The results are in line with SDT and the extended attachment perspective and indicate that students are more internally motivated to respond without prejudice if they experience relational closeness and a lack of conflict with their teacher, and more externally motivated if they have negative expectations of them. These findings are independent of students’ ethnic background, the classroom context and teachers’ anti-prejudice norms.

Despite these contributions, the value of the present study should be considered in light of some limitations and suggestions for future research. First, the study completely relied on self-reports from students, which could be affected by social desirability considerations and differences in standards of reference (Bryman, 2016). We aimed to mitigate these concerns through assessing the teacher’s anti-prejudice norm based on an aggregation of students’ perceptions of it, and in the surveys, we facilitated anonymous responding, emphasized confidentiality, and encouraged students to respond as honestly as possible.

Second, the study had a correlational design which means that no causal inferences can be drawn and that the impact of unknown confounding variables cannot be ruled out (Bryman, 2016). One important confounding variable could be parental attachment, as secure parental attachment seems to strengthen students’ prejudice regulation (de Bruijn et al., 2022) and generally predicts better relationships with teachers (Rydell et al., 2005). Future studies could control for this confound. Still, previous research found that students’ ethnic outgroup attitudes were uniquely related to the experienced closeness with their teacher but not their parents (Geerlings et al., 2017). In addition, the correlational design precludes conclusions about dynamics of student-teacher relationships and students’ RWP motivations over time, which may be particularly relevant for children, whose motivations and relationships are still developing. Longitudinal analyses could address this limitation and thereby substantially increase the implications for potential interventions as they can indicate whether changes teacher-student relationships are related to changes in students’ RWP motivations (Hoffman, 2015).

Third, we aimed to enhance the external validity compared to previous work on RWP motivations by comparing both ethnic majority and ethnic minority students in the Netherlands, but because of sample size restrictions, we had to neglect the possible differences between students with Turkish, Moroccan or Surinamese backgrounds. Relatedly, we had no information about teachers’ own ethnicity, which may have been important for student-teacher relationships (Nguyen & Le, 2023) as well as teachers’ own prejudice (Kleen et al., 2019). Still, about 90% of the teachers in the Netherlands have no migration background (Traag, 2018). Also, in contrast to other countries, students in Dutch primary education typically have one or two main teachers, and multicultural norms tend to be less pronounced compared to, for example certain North American schools (Torres & Tarozzi, 2020). Thus, for generalization purposes it is important to examine how student-teacher relationships impact RWP motivations among different groups of students and teachers in various national contexts in future research.

Yet, taken together, the results of the current study suggest that fostering relational closeness and reducing conflict and negative expectations may serve as a valuable and uncontested strategy to enhance students’ prejudice regulation. Hence, schools and policymakers aiming to promote positive intergroup relations could benefit from encouraging teachers to invest in high-quality relationships with their students.

## Supplementary Information

Below is the link to the electronic supplementary material.

Supplementary Material 1 (DOCX 28 KB)

## Footnotes

1 For readability, we refer to these participants as “students” throughout the manuscript.

2 Elementary school students in the sample typically had one or two teachers. During data collection, students were instructed to answer the questions about the teacher who was present at that time, namely the same teacher who completed the teacher survey. It is therefore unlikely that students responded about a different teacher.

3 Turks, Moroccans, and Surinamese constitute the largest non-Western groups in the Netherlands, due respectively to labor migration and the existence of former colonial ties. They are considered “typical” minorities, as they face relatively high levels of prejudice and discrimination (Andriessen, 2017).

4 Some students used the label “Frisian” under the “other” category. We treated this label as “Dutch”.
