# Supplementary material for: Elementary school students’ motivations for responding without prejudice: the role of the student-teacher relationship
Source: Soc Psychol Educ. 2026 Mar 16;29(1):31. doi: 10.1007/s11218-026-10196-z (PMC12992432; doi:10.1007/s11218-026-10196-z)
Supplement: Supplementary file 1 — Supplementary Material 1 (DOCX 28 KB) [file 11218_2026_10196_MOESM1_ESM.docx]

**Supplementary Materials**

**Table S1***Items for All Key Variables*

| *Internal RWP Motivation* |
| --- |
| *If I act nice to children from other countries or cultures, I do so. . . . . .because I want to get to know these children  . . .because I think that everyone is equal . . .because I think it is important to be nice to everyone  . . .because I like to  . . .because I find it wrong to be mean to them* |
| *External RWP Motivation* |
| *If I act nice to children from other countries or cultures, I do so. . . . . .because otherwise people might think I am a bad child . . .because I want other people to like me  . . .because other people expect me to . . .because otherwise people will think I am mean . . .because I am afraid that otherwise people will get angry at me* |
| *Closeness* |
| *I feel comfortable with my teacher. I tell my teacher things that are important to me. My teacher understands me. I think I have a good relationship with my teacher. When I have a problem, I can talk to my teacher about it. My teacher usually knows how I feel.* |
| *Negative Expectations* |
| *I wish my teacher would listen to me better when I want to say or tell something. I feel nervous when I am with my teacher. I don’t like it when my teacher gives attention to other children. I wish my teacher had more time for me. I am sometimes afraid of my teacher. I feel sad when my teacher says that I did something wrong.* |
| *Conflict* |
| *I often get into arguments with my teacher. My teacher treats me unfairly. My teacher thinks I do things secretly. I think my teacher gets tired of me in class. I can be very angry with my teacher. I feel like my teacher doesn’t trust me.*  *Note.* All items were completed by students and reflect their perspective. |

| **Table S2**  *Results of Multiple Group Confirmatory Factor Model Student-teacher Relationship (3 factors)* | | | | | | | | |
| --- | --- | --- | --- | --- | --- | --- | --- | --- |
|  | χ^2^ | df | Δ χ^2^​ | Δdf | RMSEA  (90% CI) | SRMR | CFI | TLI |
| Configural model | 499.561 | 256 |  |  | 0.042 (0.036 - 0.047) | 0.051 | 0.916 | 0.900 |
| Metric model (equal factor loadings) | 506.110 | 271 | 3.81 | 15 | 0.040 (0.034 - 0.045) | 0.054 | 0.919 | 0.909 |
| Scalar model (equal factor loadings and intercepts) | 552.311 | 286 | 45.86*** | 15 | 0.041 (0.036 - 0.046) | 0.055 | 0.909 | 0.902 |
| *Note.* Error correlations allowed between two closeness items, two conflict items, and two sets of negative expectation items. χ^2^diff based on the Satorra-Bentler scaled χ^2^ difference test. *** *p* < 0.01. | | | | | | | | |

| **Table S3**  *Results of Multiple Group Confirmatory Factor Model RWP motivations (2 factors)* | | | | | | | | |
| --- | --- | --- | --- | --- | --- | --- | --- | --- |
|  | χ^2^ | df | Δ χ^2^​ | Δdf | RMSEA  (90% CI) | SRMR | CFI | TLI |
| Configural model | 106.776 | 50 |  |  | 0.046 (0.034 - 0.058) | 0.037 | 0.971 | 0.959 |
| Metric model (equal factor loadings) | 118.351 | 57 | 10.32 | 7 | 0.044 (0.033 - 0.056) | 0.046 | 0.969 | 0.961 |
| Scalar model (equal factor loadings and intercepts) | 144.052 | 64 | 25.21*** | 7 | 0.048 (0.037 - 0.058) | 0.052 | 0.960 | 0.955 |
| *Note.* Error correlations allowed between two internal RWP motivations. Δ χ^2^ based on the Satorra-Bentler scaled χ^2^ difference test. *** *p* < 0.01. | | | | | | | | |

**Table S4**

*Model Fit Statistics for All Models*

| Model | BIC | AIC | LL(df) | Deviance ( Δ-2LL(df)) |
| --- | --- | --- | --- | --- |
| 4 (interaction with teachers’ norms) | 5773.338 | 5533.191 | -2718.596(48) | 11.008(6) |
| 3 (random slopes) | 5742.329 | 5532.200 | -2724.100(42) | 22.22(18) |
| 2 (interaction with minority versus majority) | 5677.158 | 5527.067 | -2733.533(30) | 3.356(6) |
| 1 (including predictors) | 5518.421 | 5638.495 | -2735.211(24) | 173.014(16)*** |
| 0 (intercept only) | 5699.461 | 5659.436 | -2821.718(8) |  |

*Note.*  *** = *p* < 0.001, BIC = Bayesian information criterion, AIC = Akaike information criterion, LL = loglikelihood, df = degrees of freedom.

|  | Internal Motivation | External Motivation |
| --- | --- | --- |
|  | *b (SE)* | *b (SE)* |
| *Level 1* |  |  |
| Closeness | 0.175 (0.035)** | -0.006 (0.062) |
| Negative expectations | 0.042 (0.043) | 0.324 (0.061)** |
| Conflict | -0.102 (0.052)* | -0.112 (0.080) |
| Majority vs Minority (MvM) | -0.112 (0.082) | -0.035 (0.127) |
| Gender | 0.216 (0.043)** | -0.152 (0.063)* |
| Age | -0.008 (0.030) | -0.013 (0.046) |
| *Level 1 Interactions* |  |  |
| MvM * Closeness | 0.109 (0.068) | 0.030 (0.128) |
| MvM * Negative expectations | 0.053 (0.084) | 0.011 (0.120) |
| MvM * Conflict | 0.023 (0.096) | 0.052 (0.149) |
| *Level 2* |  |  |
| Teachers’ Norms | 0.092 (0.071) | -0.150 (0.105) |
| Ethnic Composition | -0.154 (0.126) | -0.167 (0.166) |
| *Variance* |  |  |
| Level 1 | 0.494 | 0.967 |
| Level 2 | 0.009 | 0.040 |

**Table S5**
*Multilevel Regression Models with Group Interactions*

*Note. p* < 0.05, ** *p* < 0.01.

**Table S6**

*Multilevel Regression Models with Slopes and Cross-Level Interactions*

|  | Model 4 | | Model 5 | |
| --- | --- | --- | --- | --- |
|  | Internal Motivation  *b (SE)* | External Motivation  *B (SE)* | Internal Motivation  *b (SE)* | External Motivation  *b (SE)* |
| *Level 1* |  |  |  |  |
| Closeness | 0.215 (0.040)** | -0.002 (0.059) | 0.336 (0.178) | -0.307 (0.242) |
| Negative expectations | 0.049 (0.048) | 0.325 (0.068)** | 0.547 (0.165)** | 0.300 (0.287) |
| Conflict | -0.120 (0.068) | -0.074 (0.081) | -0.213 (0.227) | -0.004 (0.394) |
| Majority vs Minority | -0.115 (0.095) | -0.029 (0.134) | -0.106 (0.088) | -0.007 (0.049) |
| Gender | 0.214 (0.046)** | -0.146 (0.65)* | 0.220 (0.046)** | -0.145 (0.066)* |
| Age | -0.006 (0.053) | -0.009 (0.052) | -0.005 (0.039) | -0.031 (0.135) |
| *Level 2* |  |  |  |  |
| Teachers’ Norms (TN) | 0.092 (0.186) | -0.138 (0.128) | 0.562 (0.389) | -0.621 (0.551) |
| Ethnic Composition | -0.137 (0.175) | -0.188 (0.209) | -0.154 (0.162) | -0.204 (0.198) |
| *Cross-Level Interactions* |  |  |  |  |
| TN * Closeness | - | - | -0.048 (0.072) | 0.121 (0.094) |
| TN * Negative expectations | - | - | -0.200 (0.070)** | 0.010 (0.104) |
| TN * Conflict | - | - | 0.038 (0.089) | -0.027 (0.146) |
| *Variance* |  |  |  |  |
| Level 1 | 0.473 | 0.929 | 0.471 | 0.929 |
| Level 2 | 0.357 | 0.828 | 0.357 | 0.768 |
| Slope Closeness | 0.017 | 0.033 | 0.018 | 0.029 |
| Slope Negative expectations | 0.10 | 0.018 | 0.003 | 0.016 |
| Slope Conflict | 0.017 | 0.042 | 0.018 | 0.046 |

*Note. p* < 0.05, ** *p* < 0.01.
